# Dragonfly Hunter CZ: Mobile application for biological species recognition in citizen science

**DOI:** 10.1371/journal.pone.0210370

**Published:** 2019-01-09

**Authors:** Stanislav Ožana, Michal Burda, Michal Hykel, Marek Malina, Martin Prášek, Daniel Bárta, Aleš Dolný

**Affiliations:** 1 Department of Biology and Ecology, Faculty of Science, University of Ostrava, Ostrava, Czech Republic; 2 Institute of Environmental Technologies, Faculty of Science, University of Ostrava, Ostrava, Czech Republic; 3 Institute for Research and Applications of Fuzzy Modeling, University of Ostrava, CE IT4Innovations, Ostrava, Czech Republic; 4 Department of Informatics and Computers, Faculty of Science, University of Ostrava, Ostrava, Czech Republic; Tierarztliche Hochschule Hannover, GERMANY

## Abstract

Citizen science and data collected from various volunteers have an interesting potential in aiding the understanding of many biological and ecological processes. We describe a mobile application that allows the public to map and report occurrences of the odonata species (dragonflies and damselflies) found in the Czech Republic. The application also helps in species classification based on observation details such as date, GPS coordinates, and the altitude, biotope, suborder, and colour. *Dragonfly Hunter CZ* is a free Android application built on the open-source framework NativeScript using the JavaScript programming language which is now fully available on Google Play. The server side is powered by Apache Server with PHP and MariaDB SQL database. A mobile application is a fast and accurate way to obtain data pertaining to the odonata species, which can be used after expert verification for ecological studies and conservation basis like Red Lists and policy instruments. We expect it to be effective in encouraging Citizen Science and in promoting the proactive reporting of odonates. It can also be extended to the reporting and monitoring of other plant and animal species.

## Introduction

Citizen science is a scientific research wherein data are collected by members of the public in a voluntary capacity. Several projects relating to biodiversity research and databases are known [1]. Most of them are focused on vertebrates, especially birds [2, 3, 4]; however, the citizen-science approach has also been successfully applied for monitoring of insects such as beetles [5] and butterflies [6].

Odonates (dragonflies and damselflies) are among the best-known insect groups, and apart from butterflies, probably no other group of insects receives so much positive attention from the public [7, 8]. Conspicuous diurnal activities make odonates very easy to observe; they can be identified relatively easily because of their size and vivid colouration. Odonates are also used as flagship species and an easy-to-learn tool in environmental education programs [9]. These aspects make odonates highly suitable for monitoring projects utilising the citizen-science approach.

Despite the increasing number of citizen science projects in recent years and the knowledge that they have already provided, this practice is not fully accepted in scientific research because the data collected by the public is considered unreliable and biased [4]. Successful citizen science projects are associated with data delivered by citizen participants along with an effective control and monitoring system for ensuring data quality [3]. Therefore, there is a need for applications that help users collect data with appropriate quality using a standardised protocol. In addition, experts need to verify the collected data because in some cases, misidentification could have adverse implications for conservation policies [5].

Widespread communities of nature observers (e.g., iSpot, iNaturalist, Tela Botanica) produce outstanding collections of records. Unfortunately, these communities do not widely use new tools and techniques which could make these records more accurate and easily useful [10]. Most of them use mobile apps, but only for easier reporting while not helping users with identification of the observed organism. However, in the case of plants, semi-automated or automated identification systems which are directly used in mobile applications, such as Leafsnap, PlantNet, and Plant-O-Matic [10–12], can be found. Such systems are quite rare for animals, especially insects, and their use in mobile applications is also minimal.

In this paper, we describe a new mobile application for animal species identification and reporting through citizen science called *Dragonfly Hunter CZ*. The application represents a combination of mathematical classifiers based on fuzzy logic with a field guide for odonates found in the Czech Republic. We believe that this project will attract the public and aid in the collection of new records associated with the occurrence of odonates in the Czech Republic.

## Materials and methods

### Underlying data

**Fig 1:**
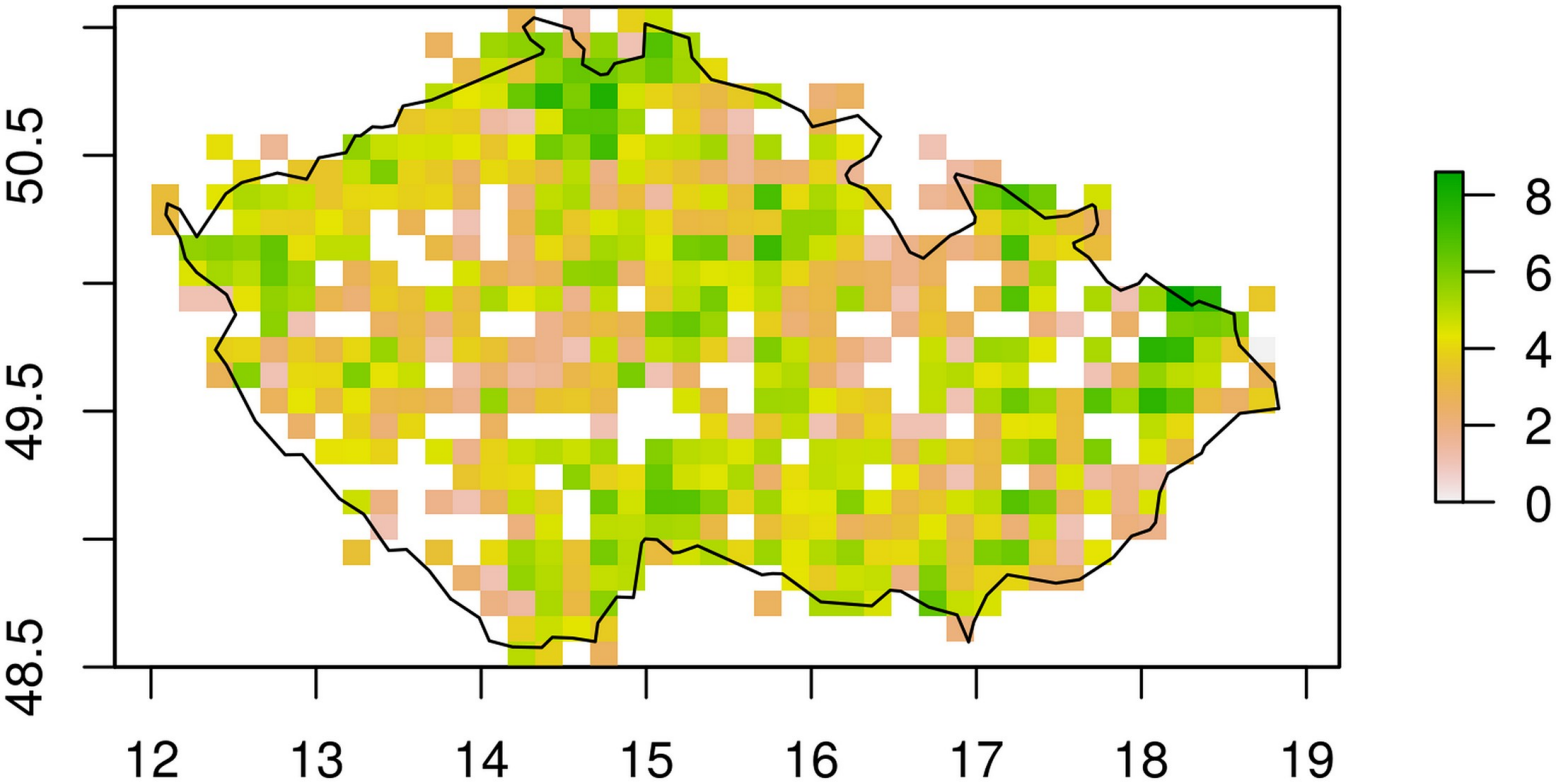
Coverage of the area of the Czech Republic by the records in the database. The map shows the number of observation records from the given area, with the legend in logarithmic scale.

The underlying mathematical model, which acts as a classifier, was developed as an expert system backed with a large database of historical and recent odonata presence records. The dataset, which is appended as a data file to this paper, consists of data used for the book ‘The Dragonflies of the Czech Republic: Ecology, Conservation and Distribution’ [13], data from the Species Occurrence Database run by the Nature Conservation Agency of the Czech Republic [14], and the personal records of the authors (see also Fig 1). The database has records dating back to 1850 with basic information about the species distribution. It consists of verified data collected by amateur and professional odonatologists, which were used for two books on the odonata species of the Czech Republic [13, 15]. The database contains full names of the observed species, date of the observation, location, altitude, biotope (the 15 types), and the number of specimens observed. Records from the year 2002 and later were selected for further processing. A stratified split with respect to the species name was performed on the dataset in order to obtain training and testing datasets of size 74567 and 18570 records, respectively (see S1 Appendix).

As the data do not contain information about colours, colours had to be generated for testing purposes. Based on expert knowledge, up to three possible colours were randomly selected and assigned to each record in the database to simulate a real behaviour of a user. Being aware of possible bias introduced by that process, the number of randomly selected colours was reduced to three, although the odonata species may have more colours.

As the creation of the classification model is fully automated, it will be easy to re-fit it on a regular basis to reflect the most recent data collected by the system and provide it to the users as an update.

### Mathematical classifier

As the historical and recent data do not contain all the information needed, the classification model is created as an expert system that uses expert knowledge to perform the automatic classifications. Once new data with all the needed attributes are collected from users, the expert system can be replaced with a classifier constructed using machine learning. The expert knowledge, formulated by professional odonatologists for each of the 74 species, comprises the taxonomical suborder (Anisoptera, Zygoptera), seasonality (days of the year during which the species can probably be observed), altitudes and biotopes preferred by the species, and colours assigned separately for males/females: yellow, orange, red, green, blue, brown, and black.

The information about location (encoded for a network of ca. 11.2 × 12 km^2^ areas) and commonality were the only attributes used directly from the training data during the creation of the classification model.

The expert information was encoded in the form of *fuzzy sets*. A fuzzy set [16] is a generalisation of a classical set, which allows an element to be a member of a set only partially, as described by a degree in the interval [0, 1], where ‘1’ represents full membership, ‘0’ represents non-membership, and intermediate values denote different intensities of membership.

For instance, experts defined a fuzzy set of days of the year for each species: ‘1’ was assigned to days of the year with a high probability of occurrence, and ‘0’ to days when the probability of occurrence was nil. Grades between ‘0’ and ‘1’ were assigned to borderline days in order to soften the transitions.

Based on the expert knowledge provided as fuzzy sets, the classifier uses the information provided by the user (or his/her mobile device) to sort the males or females of the odonata species in decreasing order of relevance.

Specifically, the relevance of odonata species *o* for a given user input is computed as

commonality(o)×suborder(o)×colour(o)×position(o)×altitude(o)×biotope(o)×season(o),

where each multiplicand is a coefficient from the interval [0, 1] obtained as follows.

*Commonality* is determined for each species *o* from the underlying training dataset *D* as

$$commonality(o)=\sqrt{\frac{\#o}{\#max}},$$

where #o and #max denote the number of records of species *o* and the number of records of the most frequent species, respectively.

*Suborder(o)* is simply equal to ‘0’ or ‘1’ depending on whether the species *o* is of the correct suborder, as specified by the user.

*Colour(o)* is obtained by using the technique of *compositions of fuzzy relations* [17], as follows:

$$colour=\text{min}\left\{0.7{\;(in^{T}S)}^{}◦A+0.3,\;in^{T}◦E\right\},$$

where ***in*** is a binary input vector of colours specified by the user, *S* is a matrix encoding similar colours so that even the colours not selected by the user gain a small influence based on their similarity to the selected colour, *A* is a numeric matrix, which assigns to each species a colour that increases the relevance additively, and *E* is a numeric matrix that assigns to each species a colour whose occurrence prevents the species from being highly relevant. In other words, *A* and *E* specify the colours that the species may and must not have, respectively. The ◦ operator represents the composition of fuzzy relations. The influence of the additive part of the composition is slightly shifted upwards by multiplying the results with 0.7 and adding 0.3.

*Position(o)* is obtained by looking up the table of the presence of odonata species in the given region (ca. 11.2 × 12 km^2^ area corresponding to the given GPS coordinates). The species that are present in the region are assigned ‘1’, whereas those that are absent are assigned 0.5. The presence of odonates was obtained from the training dataset.

Similarly, *biotope(o)* gets a value from the interval [0.8, 1] according to the expected appearance of the species in the biotope, which is determined from the underlying training dataset.

The last two coefficients, *season(o)* and *altitude(o)*, obtain a value from the interval [0.5, 1] based on expert knowledge on seasonality and preferred altitude of the species, modelled using fuzzy sets with a trapezoidal shape.

As all information provided by the user is optional, the multiplicand, which relies on the user input that was not provided, is assigned the value of ‘1’.

### Application development

The application was developed using the open-source framework NativeScript that uses the JavaScript programming language, which is commonly used to develop mobile applications that are portable to different platforms. The server side is powered by Apache Server with PHP and a MariaDB SQL database.

## Results

### Application details

**Fig 2:**
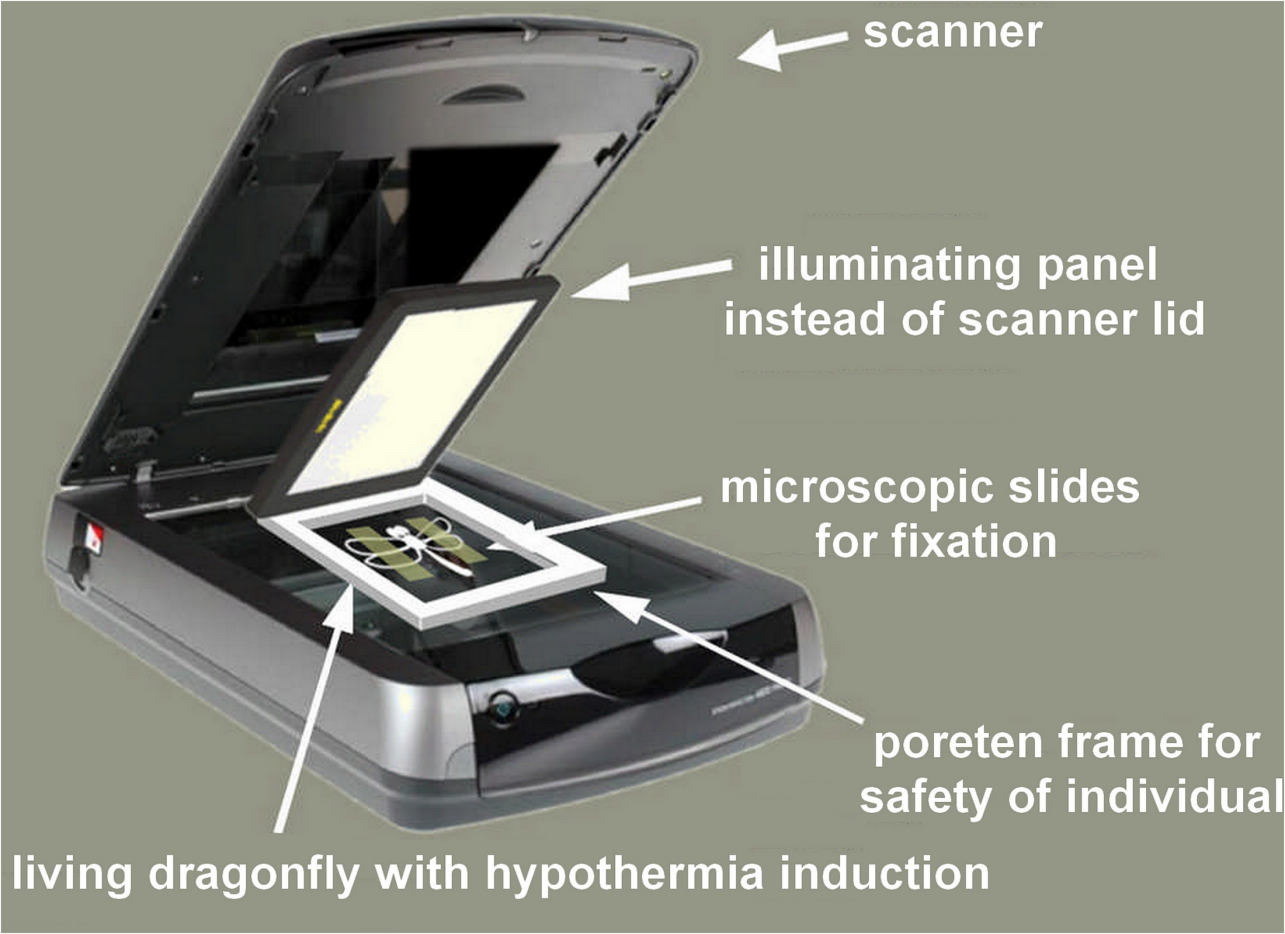
Method used to scan a living specimen with a desktop scanner.

*Dragonfly Hunter CZ* is a free Android application that contains a list of all 74 odonata species recorded in the Czech Republic (freely available at https://play.google.com/store/apps/details?id=org.nativescript.DragonflyRecognizer; the source codes are available at https://gitlab.com/Olomer/dragonfly-hunter-cz). The application provides a detailed description and pictures of both the male and female of the species. Living specimens of all species of both genders were photographed by scanning using a table scanner (Fig 2), all of which are presented in vivid colour images. *Dragonfly Hunter CZ* provides a system for species classification based on observation details such as date, GPS coordinates and altitude, biotope type, taxonomical suborder (Anisoptera, Zygoptera), and colouring. In odonates, the colouration of males and females differ significantly; therefore, the classification algorithm differentiates between the genders.

**Fig 3:**
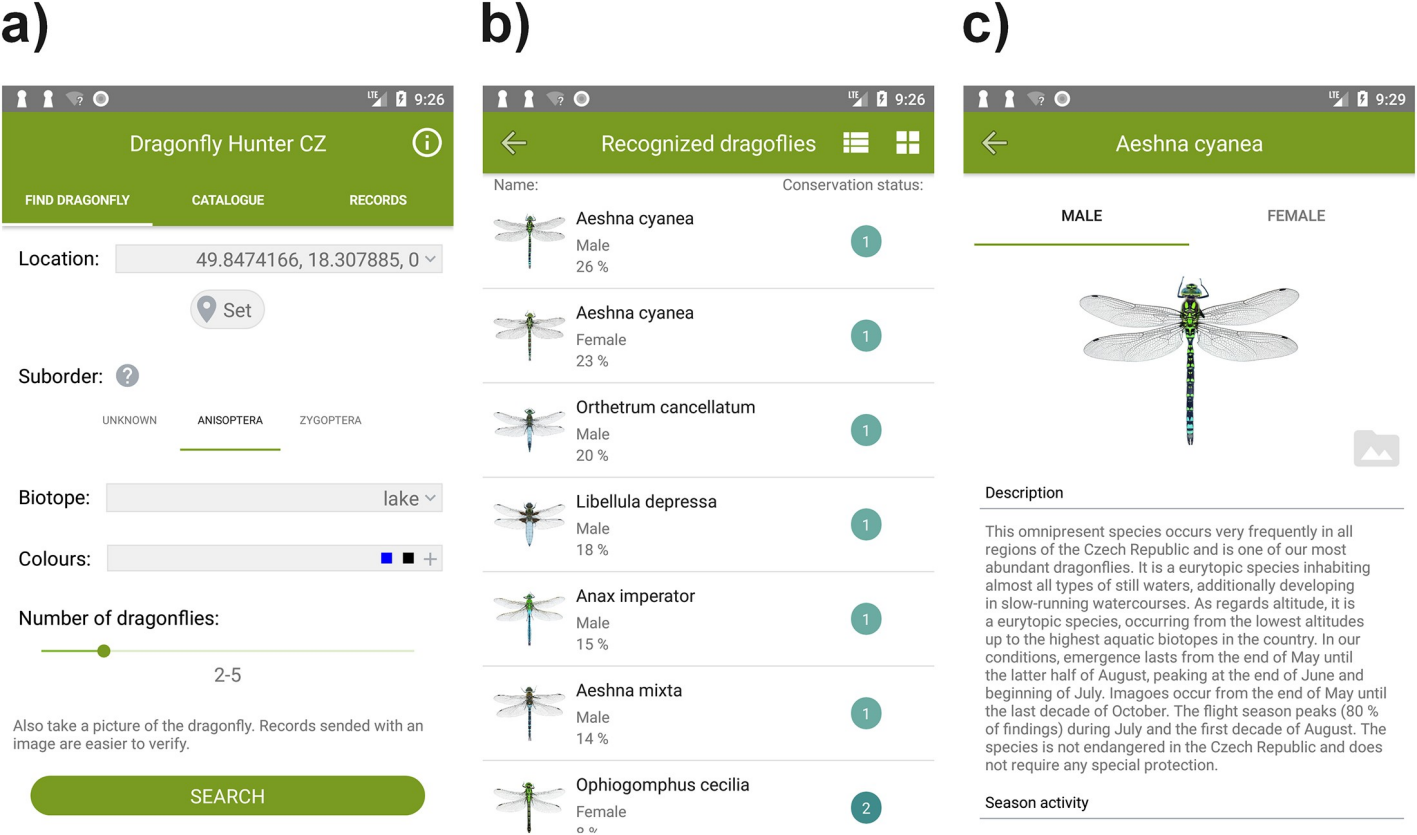
Sample screens from the application. a) interface of the classification algorithm, b) catalogue of odonates, c) information about the species.

Each category of information is optional, as the classifier can work with incomplete information. The result is a list of species based on their relevance to the given search criteria, with the male and female of the species listed separately. The search criteria were chosen considering their ease of comprehensibility by amateurs. The algorithm works offline, i.e., no internet connection is needed for the classification of the species. In addition to classification, the application allows the user to participate in monitoring the presence of odonates by sending a report of the observation to the application servers. After the list of relevant species is shown based on user-provided information, the user may select the appropriate specimen (also by comparing the observed individual with provided photographs) and send an observation report to a central server. Additionally, the application provides a list of reports, and allows the user to add a photograph of the observed specimen, and to manage the reports (edit, delete). The observation reports are assigned to the user’s identity provided by the Android OS. All the attributes provided by the user to classify the observed odonates are stored as they may serve in verification of the record, to prepare a new version of the classification model, or provide other valuable information to researchers in the future. The information collected for both reports and classifications are as follows (Fig 3):

- Date of observation—this attribute is set to the current date by default, and indicates the seasonality of the species;
- Location and altitude—obtained automatically from the GPS module of the device;
- Suborder—Anisoptera or Zygoptera; detailed descriptions and figures of both suborders are provided;
- Biotope—the user can select one of the following options to further describe the place of observation: lake, puddle, canal (artificial canals and drainage channels, ditches), garden pool, quarry (pools in quarries and gravel pits), wetland (temporary pool), brook, headwaters and spring, organic soil wetland (bog, fen), pond, river, oxbow lake, reservoir in valley, and terrestrial forest or non-forest habitat;
- Colour—up to three observed colours: yellow, orange, red, green, blue, brown, or black;
- Abundance—number of observed specimens: 1, 2–5, 6–10, 11–20, 21–50, 51–100, or 100+;
- Photograph—optional.

All user-added observation reports are subject to manual verification by expert odonatologists, and the user is automatically notified about the result of the verification process.

The entire application is bilingual, depending on the language of mobile device. If the user uses the Czech language, the application is also in Czech, for every other language the application operates in English.

### Classification accuracy

Based solely on very easily detectable descriptions such as colour, location, and date, the classification model is highly accurate, easily understandable, and extendable with possible future improvements. The classification model orders the species/sex pairs (148 classes in total) in descending order by relevance. Specifically,

- the correct classification is on average at position 4.78 (median: 3^rd^ position) among 148 classes;
- the correct classification is on the first screen of the display (five items per screen) with probability 0.69;
- 95% of test cases result in correct classification within the first 15 places (three screens of results).

**Fig 4:**
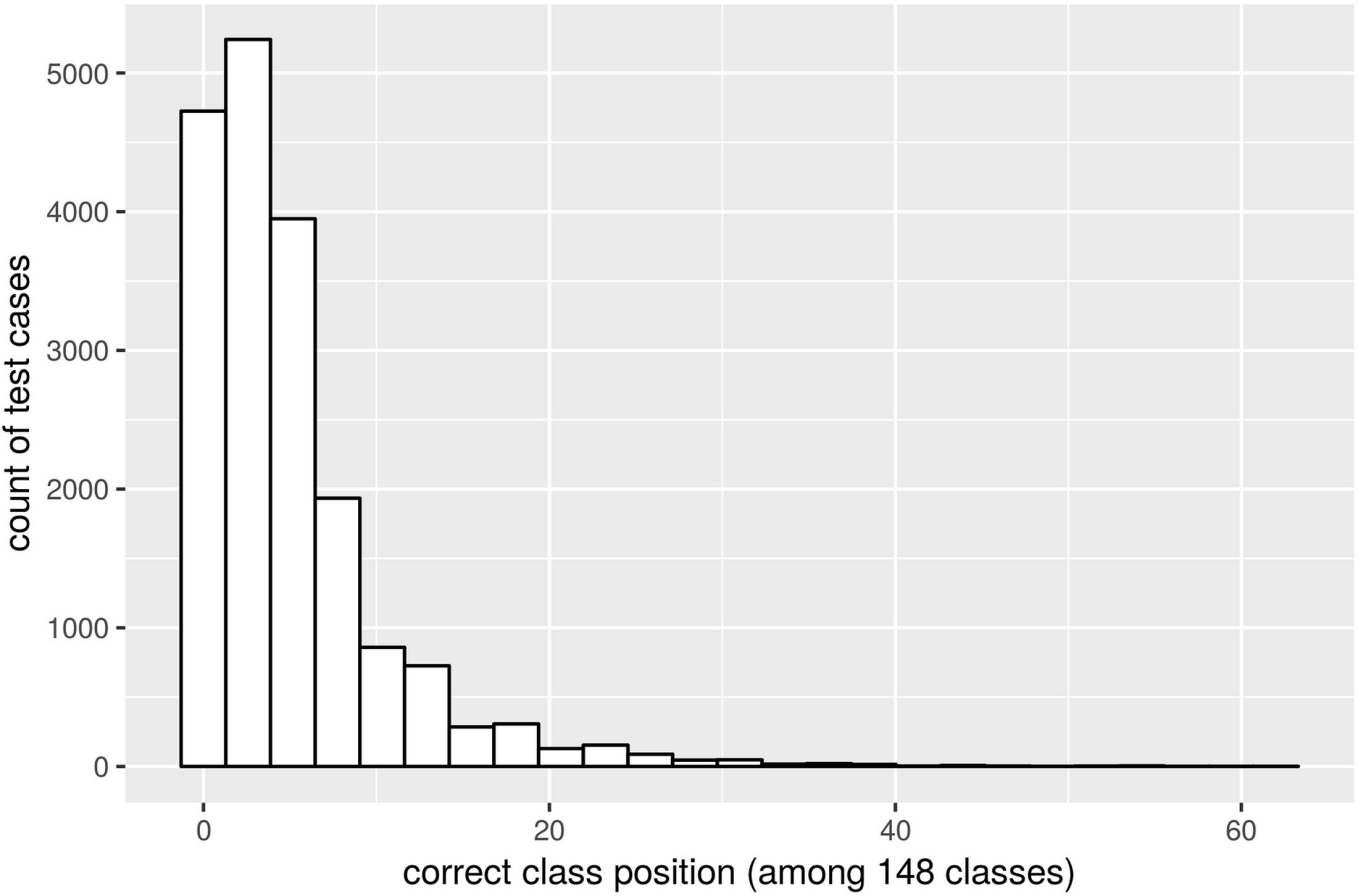
Histogram of the positions of correct classifications in a list sorted by relevance (evaluated on the testing dataset).

A histogram of the positions of correct classifications is presented in Fig 4. All these results were obtained on the testing dataset; that is, from those parts of the data that were not used to develop the model.

When the sex of the observed individual is ignored, the classification model finds the correct classification on average at position 3.49 (median: 2^nd^ position) among 74 classes and 95% of the test cases result in correct classification within the first 10 places.

To mitigate the risk of temporal changes bias within the testing of the model, an experimental fit of the model was also conducted on data not containing the last three years (70688 records), which were left for testing (22449 records). The average position of the correct classification within the ordered list of classes slightly decreased to 5.32 with 95% probability of a correct answer to be on the position between the 1st and 17th place of 148.

### Application database

**Fig 5:**
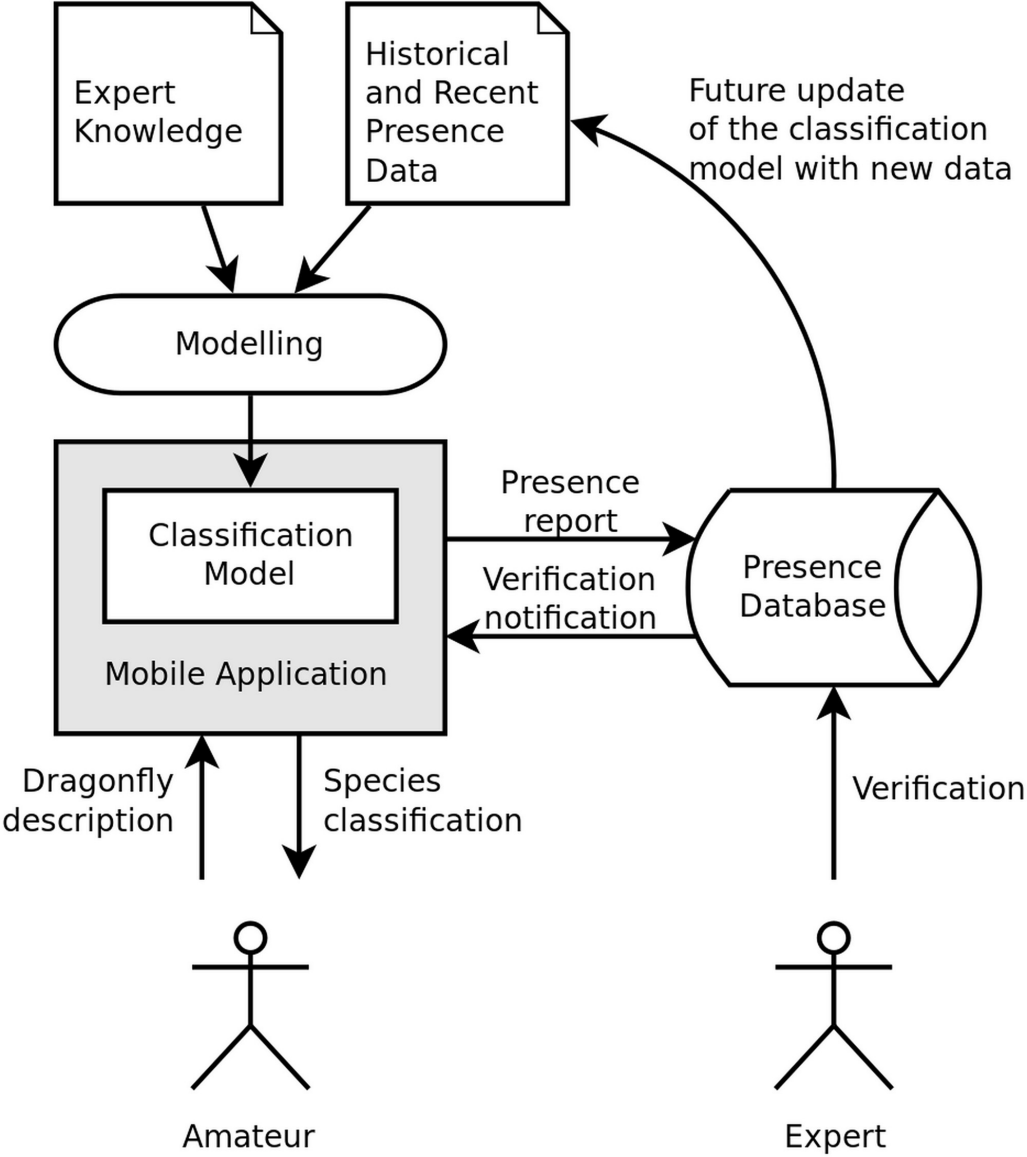
Operation of the mobile application. The application’s classification model is based on historical and recent presence data and expert knowledge. The amateur user inputs observed dragonfly description into the application and obtains a list of probable species. The user then selects the most appropriate classification from the list (possibly also by comparing the observed species with the photograph) and sends the observation report to the central database, where the expert biologist verifies the records and the amateur user is notified. Stored presence records will be used to update the classification model after each year in the future.

All the input information that the user chooses in our classification system are stored together with the record of the species. This information can then be used to correct the verification of the record by the experts on the Czech odonates. Each record is manually verified—even though this process may seem complicated, it is generally used for the national species databases of the Czech Republic, with only a few verifiers necessary for hundreds or thousands of records per year. Additionally, verified records will be used each year to update the classification system (the operation of the overall system is shown in Fig 5). Our database is also open source, which means that at the end of each year all records are sent to the Species Occurrence Database and publicly shared on our server with everyone interested in odonatology.

## Discussion

### Importance of the application

Odonata have strong potential as a model group of organisms for testing the cost-effectiveness of citizen-science projects because of their popularity within the scientific community and the general public, but mainly for their use as indicators in assessing a habitat quality. The life cycle of odonates involves a larval aquatic stage and an adult terrestrial stage; the need for both types of habitats and their sensitivity to man-made habitat changes has made them suitable bio-indicators of both terrestrial and aquatic environments and they are often used in this capacity [18–21]. Therefore, they are widely considered as good environmental indicators to assess the ecological health and human-induced habitat changes as a consequence of intensive management, urbanisation, agriculture, mining, military activity, and intensive fish farming [22–24].

Our application enables both professionals and amateur odonatologists to report records on odonate occurrences in the entire country by a simple and effective method. Finally, we designed this application in the hope that it will enhance public interest in odonatology and nature in general. *Dragonfly Hunter CZ* is a fast and accurate way to obtain data, which can be used after expert verification for various ecological studies and conservation artefacts such as Red Lists and policy instruments. Thus, this application may provide very valuable information for documenting a species’ presence, including newly discovered or rediscovered species. Nowadays in particular, when most smartphones have a camera sufficient for taking high-resolution images [25], in the case of rediscovered species, the associated good-quality images should suffice, as illustrated by the example of the reappearance of presumed extinct lilypad whiteface (*Leucorrhinia caudalis*) in the Czech Republic [26]. Dragonflies do not belong to the groups of animals that are difficult to identify from macroscopic morphology and photographing can be used to reliably identify them, unlike many others; for example, a large proportion of the world’s marine animals [27].

### Building the model

Initially, we attempted to find a classification model based on machine learning. Because of the need for good handling of missing values (all user input values are optional), we selected the popular classification and regression trees algorithm (CART) implemented in the *rpart* package for R [28]. The classification task using automated machine learning methods was very challenging mainly because of a very large set of target classes (74 species × 2 genders = 148 classes). Moreover, although the results were highly accurate from the statistical point of view, the actual user experience was not satisfactory. Human experts very often found the results confusing and non-intuitive. Therefore, we directed our efforts towards building an expert system based on the compositions of fuzzy relations [17]. This model shows more concise and intuitive results by sorting the species in an order similar to the expectations of human experts.

### Application benefits

The benefits of the mobile application are two-fold. Firstly, the application helps in classification of odonata species and thus propagates ecology and nature conservation. Secondly, the application enables amateurs to be involved in science and research by observing and reporting the presence of species.

The classification model is based on a combination of an expert system and information obtained from existing historical and recent data about the occurrence of odonates in the Czech Republic. In the future, the data collected by amateurs can be used to further enhance the classifier, for instance, by enabling frequency analysis on how users assign colours to odonatan species, or incorporation of the most recent occurrences of the species into the model. The proposed framework may also be quite easily adapted for other organisms.

Based on our experience, the application of fuzzy logic, which facilitates the use and capture of vague notions, enabled expert biologists to explicitly state their knowledge in a natural manner, similar to natural language. A good example of using fuzzy logic can be found in conservation biology [29, 30], whereas it is minimal in animals, especially in insects. Furthermore, the co-operation of biologists with mathematicians brought new insights and ideas to further develop tools for fuzzy modelling.

At first, we discussed the kind of technology that could be used to develop our application. We are a small team; therefore, rather than develop two different applications for both currently popular mobile operating systems, we decided on a multi-platform solution. We searched for such a solution which could access as many native components as possible (GPS, camera, etc.). A great advantage is the use of open-source framework NativeScript because developers can create these components by themselves [31]. With this framework we could create a single application and then build it natively for different operating systems, such as Android or iOS. Furthermore, creating a NativeScript application is much easier as we could focus and add functionalities which had not been planned at the start of the development. For example, saving an observation for later online evaluation was a function we had thought was impossible in the first iteration of the development. Currently, our mobile application supports only the Android operating system; however, an iOS version is being developed. The application can also be relatively easily transformed into a web application (same programming language), which we are also planning to develop this year.

### Future vision

*Dragonfly Hunter CZ* is our initial project, which represents the evolution of information technology in the field of biomonitoring. We are considering integrating our system with automatic image recognition based on pattern recognition or deep learning. These are among the key methods that will be used in the future, not just for their usefulness but also for their conservation potential. Using mobile devices and such methods can provide essential tools which could even save some endangered wild species from overcollection. Similarly, well sorted and publicly open databases of records and images of the species should be a general idea for such citizen science projects [25, 32], which is also our intention. We also plan to port the application to other operating systems such as iOS. A natural extension planned is the *Dragonfly Hunter EU* as a citizen-science application for Odonata in the entire Europe. In addition to dragonflies, the application may be very easily extended to the identification and reporting of other species of animals and plants.

## Supporting information

S1 Appendix The training and testing datasets used for the classification model. (XLSX)

## References

[1] Kobori H, Dickinson JL, Washitani I, Sakurai R, Amano T, Komatsu N, et al Citizen science: A new approach to advance ecology, education, and conservation. Ecol Res. 2016;31: 1–19. 10.1007/s11284-015-1314-y

[2] Dickinson JL, Shirk J, Bonter D, Bonney R, Crain RL, Martin J, et al The current state of citizen science as a tool for ecological research and public engagement. Front Ecol Environ. 2012;10: 291–297. 10.1890/110236

[3] Sullivan BL, Aycrigg JL, Barry JH, Bonney RE, Bruns N, Cooper CB, et al The eBird enterprise: An integrated approach to development and application of citizen science. Biol Conserv. 2014;169: 31–40. 10.1016/j.biocon.2013.11.003

[4] Bonney R, Shirk JL, Phillips TB, Wiggins A, Ballard HL, Miller-Rushing AJ, et al Citizen science: Next steps for citizen science. Science. 2014;343: 1436–1437. 10.1126/science.1251554 24675940

[5] Gardiner MM, Allee LL, Brown PMJ, Losey JE, Roy HE, Smyth RR. Lessons from lady beetles: Accuracy of monitoring data from US and UK citizen science programs. Front Ecol Environ. 2012;10: 471–476. 10.1890/110185

[6] Devictor V, Whittaker RJ, Beltrame C. Beyond scarcity: Citizen science programmes as useful tools for conservation biogeography. Divers Distrib. 2010;16: 354–362. 10.1111/j.1472-4642.2009.00615.x

[7] Kalkman VJ, Clausnitzer V, Dijkstra KDB, Orr AG, Paulson DR, Van Tol J. Global diversity of dragonflies (Odonata) in freshwater. Hydrobiologia. 2008;595: 351–363. 10.1007/s10750-007-9029-x

[8] Barua M, Gurdak DJ, Ahmed RA, Tamuly J. Selecting flagships for invertebrate conservation. Biodivers Conserv. 2012;21: 1457–1476. 10.1007/s10531-012-0257-7

[9] Clausnitzer V, Simaika JP, Samways MJ, Daniel BA. Dragonflies as flagships for sustainable use of water resources in environmental education Dragonflies as flagships for sustainable use of water resources in environmental education. Appl Environ Educ Commun. 2017 10.1080/1533015X.2017.1333050

[10] Joly A, Goëau H, Glotin H, Spampinato C, Bonnet P, Vellinga W-P, et al LifeCLEF 2016: Multimedia life species identification challenges In: Fuhr N, Quaresma P, Gonçalves T, Larsen B, Balog K, Macdonald C, et al, editors. Experimental IR Meets Multilinguality, Multimodality, and Interaction. Cham: Springer International Publishing; 2016 pp. 286–310.

[11] Kumar N, Belhumeur PN, Biswas A, Jacobs DW, Kress WJ, Lopez IC, et al Leafsnap: A computer vision system for automatic plant species identification In: Fitzgibbon A, Lazebnik S, Perona P, Sato Y, Schmid C, editors. Computer Vision—ECCV 2012. Berlin, Heidelberg: Springer Berlin Heidelberg; 2012 pp. 502–516.

[12] Goldsmith GR, Morueta-Holme N, Sandel B, Fitz ED, Fitz SD, Boyle B, et al Plant-O-Matic: A dynamic and mobile guide to all plants of the Americas. Methods Ecol Evol. 2016;7: 960–965. 10.1111/2041-210X.12548

[13] Dolný A, Bárta D, Waldhauser M, Holuša O, Hanel L. The Dragonflies of the Czech Republic: Ecology, Conservation and Distribution. Vlašim: ČSOP; 2007.

[14] Nature Conservation Agency CR 2016. Nálezová databáze ochrany přírody / Species Occurrence Database. [on-line databáze; portal.nature.cz]. [cit. 2016-01-07]

[15] Dolný A, Harabiš F, Bárta D. Vážky (Isecta: Odonata) České republiky. Praha: Academia; 2016.

[16] Zadeh LA. Fuzzy sets. Inf Control. 1965;8: 338–353. 10.1016/S0019-9958(65)90241-X

[17] Cao N, Štěpnička M, Burda M, Dolný A. Excluding features in fuzzy relational compositions. Expert Syst Appl. 2017;81: 1–11. 10.1016/j.eswa.2017.03.033

[18] Simaika JP, Samways MJ. An easy-to-use index of ecological integrity for prioritizing freshwater sites and for assessing habitat quality. Biodivers Conserv. 2009;18: 1171–1185. 10.1007/s10531-008-9484-3

[19] Dolný A, Harabiš F, Bárta D, Lhota S, Drozd P. Aquatic insects indicate terrestrial habitat degradation: Changes in taxonomical structure and functional diversity of dragonflies in tropical rainforest of East Kalimantan. Trop Zool. 2013;25: 141–157. 10.1080/03946975.2012.717480

[20] Gerlach J, Samways M, Pryke J. Terrestrial invertebrates as bioindicators: An overview of available taxonomic groups. J Insect Conserv. 2013;17: 831–850. 10.1007/s10841-013-9565-9

[21] Bried JT, Samways MJ. A review of odonatology in freshwater applied ecology and conservation science. Freshwater Science 2015;34(3): 1023–1031. 10.1086/682174

[22] Samways MJ, Steytler NS. Dragonfly (Odonata) distribution patterns in urban and forest landscapes, and recommendations for riparian management. Biol Conserv. 1996;78: 279–288. 10.1016/S0006-3207(96)00032-8

[23] Dolný A, Harabiš F. Underground mining can contribute to freshwater biodiversity conservation: Allogenic succession forms suitable habitats for dragonflies. Biol Conserv. 2012;145: 109–117. 10.1016/j.biocon.2011.10.020

[24] Harabiš F, Dolný A. Military training areas as refuges for threatened dragonfly species: Effect of spatial isolation and military activity. Biol Conserv. 2018;217: 28–35. 10.1016/j.biocon.2017.10.021

[25] Minteer BA, Collins JP, Love KE, Puschendorf R. Ecology. Avoiding (re)extinction. Science. 2014;344: 260–261. 10.1126/science.1250953 24744362

[26] Dolný A, Waldhauser M, Kvita L, Kocourková L. New Records of Lilypad Whiteface Leucorrhinia Caudalis (Odonata: Libellulidae) in the Czech Republic. Acta Musei Silesiae, Sci Nat. 2014;63: 185–192. 10.2478/cszma-2014-0019

[27] Minteer BA, Collins JP, Puschendorf R. Specimen collection: An essential tool—Response. Science (80-). 2014;344: 816 10.1126/science.344.6186.816-a 24855247

[28] Therneau T, Atkinson B, Ripley B. rpart: Recursive Partitioning and Regression Trees. R package version 4.1–10. 2015. https://CRAN.R-project.org/package=rpart

[29] Cheung WWL, Pitcher TJ, Pauly D. A fuzzy logic expert system to estimate intrinsic extinction vulnerabilities of marine fishes to fishing. Biol Conserv. 2005;124: 97–111. 10.1016/j.biocon.2005.01.017

[30] Kovac M, Grošelj P. Toward objective assessment of the conservation status of (the Natura 2000) forest habitat types: A comparison of a qualitative and a quantitative modeling approach. Ecol Indic. 2018;89: 281–289. 10.1016/j.ecolind.2018.02.001

[31] Vilcek T, Jakopec T. Comparative analysis of tools for development of native and hybrid mobile applications. 2017 40th Int Conv Inf Commun Technol Electron Microelectron. 2017; 1516–1521.

[32] Grandcolas P. Loosing the connection between the observation and the specimen: A by-product of the digital era or a trend inherited from general biology? Bionomina. 2017;12: 57 10.11646/bionomina.12.1.7

